# Association Mapping for Fiber-Related Traits and Digestibility in Alfalfa (*Medicago sativa*)

**DOI:** 10.3389/fpls.2016.00331

**Published:** 2016-03-18

**Authors:** Zan Wang, Haiping Qiang, Haiming Zhao, Ruixuan Xu, Zhengli Zhang, Hongwen Gao, Xuemin Wang, Guibo Liu, Yingjun Zhang

**Affiliations:** ^1^Institute of Animal Sciences, Chinese Academy of Agriculture SciencesBeijing, China; ^2^Institute of Dry Farming, Hebei Academy of Agriculture and Forestry SciencesHengshui, China; ^3^Department of Grassland Science, China Agricultural UniversityBeijing, China

**Keywords:** alfalfa, association mapping, fiber-related traits, Simple sequence repeat (SSR)

## Abstract

Association mapping is a powerful approach for exploring the molecular genetic basis of complex quantitative traits. An alfalfa (*Medicago sativa*) association panel comprised of 336 genotypes from 75 alfalfa accessions represented by four to eight genotypes for each accession. Each genotype was genotyped using 85 simple sequence repeat (SSR) markers and phenotyped for five fiber-related traits in four different environments. A model-based structure analysis was used to group all genotypes into two groups. Most of the genotypes have a low relative kinship (<0.3), suggesting population stratification not be an issue for association analysis. Generally, the Q + K model exhibited the best performance to eliminate the false associated positives. In total, 124 marker-trait associations were predicted (*p* < 0.005). Among these, eight associations were predicted in two environments repeatedly and 20 markers were predicted to be associated with multiple traits. These trait-associated markers will greatly help marker-assisted breeding programs to improve fiber-related quality traits in alfalfa.

## Introduction

Alfalfa (*Medicago sativa*) is one of the most important forage crops in the world due to its high biomass and choice nutritional profiles and it provides reliable sources of protein and minerals to animals. The main objective, however, in many alfalfa quality breeding programs is to improve the digestibility (Buxton and Redfearn, 1997) for poor stem digestibility would cause major loss in animal feeding values (Mowat et al., 1965). A research indicated minor improvement in alfalfa stem digestibility would impact agriculture economically (Jung and Allen, 1995). In these sense, efforts in traditional breeding of improving the quality traits as well as yield, resistance, and agronomic traits are necessary. The feeding quality traits are usually quantitative, i.e., controlled by multiple genes. Understanding the genetic architecture of these traits on the molecular level is of necessity for efficient molecular breeding.

Currently, linkage analysis via QTL mapping, genome-wide association mapping (GWAS), and joint-mapping by combining linkage and association analysis (Ed Buckler's NAM population in maize) are the three main methods for dissecting complex quantitative traits. Compared with the traditional linkage analysis based on mapping populations, association mapping, has been proposed as an alternative powerful tool to overcome limitations of pedigree based QTL mapping for it has higher mapping resolution, reduced research time, and greater allele number (Zhu et al., 2008). By utilizing historical recombinations that break LDs (linkage disequilibrium), association mapping has been widely adopted for almost all major crop species for gene identification and QTL validation, as well as better understanding of the genetic basis of complex traits (Gupta et al., 2011; Jiang et al., 2014; Wei et al., 2014; Font i Forcada et al., 2015; Portis et al., 2015). Given the facts that the most forage plants have a short selection and breeding history, Li et al. (2011) performed a GWAS analysis to map the yield and stem composition in an alfalfa breeding population of 190 individuals based on 71 SSR markers, and identified only one SSR strongly associated with acid detergent fiber (ADF) and acid detergent lignin (ADL), respectively. In this study, the association analysis was conducted for five fiber-related traits using a panel of 336 alfalfa genotypes, partially derived from the alfalfa core collection set developed by Basigalup et al. (1995) plus some germplasm from China. The study aimed to identify desirable alleles which could show significant trait-marker associations for the improvement of digestibility in alfalfa.

## Materials and methods

### Plant materials and experimental design

A total of 336 cultivated tetraploid alfalfa genotypes from 75 *M. sativa* subsp. *sativa* accessions were selected to construct the association mapping population (Table S1). Each accession was represented by four genotypes, except for the Chinese accessions represented by eight genotypes for each accession. Nine Chinese accessions were collected from National Herbage Germplasm Bank of China; two accessions from Syria, one from Libya, and one accession from Sudan provided by the Institute of Animal Science, Chinese Academy of Agricultural Science (Beijing, China); the rest 62 accessions were provided by USDA National Plant Germplasm System (NPGS). All genotypes were grown and clonally propagated. Field experiments were conducted at the experimental station at the Institute of Dry Farming, Hebei Academy of Agriculture, and Forestry Sciences (Hengshui, Hebei Province, 37°44′N; 115°42′E) in May 2012, and at the experimental station of Institute of Animal Science of CAAS (Changping, Beijing, 40°10′N; 116°13′E) in May 2014. The experiment at each location used a randomized completed block design with two replications, each of which contained six clones. Within each plot, each cloned plant was spaced by 30 cm and each row was spaced by 75 cm. The biomass above the ground were trimmed ~1 month after establishment and then regrew for the remainder of the season.

### Phenotyping

Plants were tested under four environments (Hengshui, 2013, 2014, 2015; Changping, 2014, designed as 13HS, 14HS, 15HS, and 14CP, respectively). Plant leave tissue samples were ground and then passed through a 1-mm mesh screen (Cyclone Mill, UDY Mfg., Ft. Collins, CO). Aliquots of each sample were scanned by near-infrared reflectance spectroscopy. Measurements were obtained using a FOSS 5000 scanning monochromator (FOSS, Denmark) and recorded at 2-nm intervals between 1100 and 2500 nm. A subset of 100 samples was selected for the calibration of spectroscopy using chemical analyses. Coefficients of determination (*R*^2^) were 0.9317 for ADF, 0.9634 for neutral detergent fiber (NDF), 0.6769 for ADL, 0.4950 for NDF digestibility (*in vitro* 30 h, NDFD 30 h), and 0.9001 for NDFD 48 h.

### Genotyping

Eighty-five polymorphisms SSR primers (Eujayl et al., 2004; Robins et al., 2007) were used for genotyping. DNA extraction, PCR amplification, electrophoresis, and SSR genotyping analysis were conducted according to the methods described by Qiang et al. (2015).

### Data analyses

Analysis of variance (ANOVA) of all phenotypic data based on the means of traits of each accession under four environments was conducted as model: Phen = genotypes + environments + e. where Phen as the phenotypic observation, genotypes as the genetic effect, environments as the effect of the four environments, and e as the residual. All analyses were performed using SAS 8.02 (SAS Institute, 1999). Broad-sense heritability (*H*^2^) was calculated as the genotypic variance divided by the total variance.

A kinship matrix was calculated using SPAGeDi software (Hardy and Vekemans, 2002).

The association between the phenotypes and markers was performed using Tassel v2.1 software (Bradbury et al., 2007). Three models were tested, namely the simple general linear model (GLM, Naive-model), the structured association model (GLM, Q model), and the mixed linear model (MLM, Q + K model) (Yu et al., 2006). The marker-trait association was considered as significant using a threshold of *P* < 0.005.

## Results

### Phenotypic variation

The descriptive parameters of the five measured traits under four environments were shown in Table 1. In summary, the ADF ranged from 20.59 to 42.85%, with an average of 31.22–33.63%; the NDF ranged from 25.08 to 53.06% with a mean of 36.72–42.89%; the NDFD 30 h alternated from 12.28 to 26.75% with an average of 16.68–23.81%; the NDFD 48 h ranged from 11.02 to 22.4% with an average of 15.47–17.55%; the ADL varied from 2.70 to 8.66% with an average of 4.26–6.56%. All the datasets showed a normal or nearly normal distribution (Figure S1). The broad-sense heritability of most traits was relatively high, ranging from 63% for NDF 30 h to 76% for NDF 48 h (Table 1), except for the heritability of ADL (45.1%), indicating the majority of studied traits were dominated by the genetic factors rather than the environmental variations. All five traits were significantly influenced by genotypes, environments and genotype × environments interactions (Table 1).

**Table 1:** **Phenotypic variation for five traits in alfalfa in four environments**.

**Traits**	**E**	**Min**	**Max**	**Mean /± SD**	***H*^2^**	**G**	**E**	**G × E**
ADF (DM %)	13HS	25.3	41.9	33.63±2.85	73.6	^***^	^***^	^***^
	14HS	21.3	42.9	33.00±3.32				
	15HS	20.6	40.1	31.80±2.85				
	14CP	22.8	39.0	31.22±2.69				
NDF (DM %)	13HS	31.5	53.1	42.89±3.60	74.6	^***^	^***^	^***^
	14HS	25.1	50.6	38.74±3.82				
	15HS	25.5	45.6	36.72±3.24				
	14CP	28.4	46.8	38.11±3.10				
NDFD 30 h (NDF %)	13HS	12.3	21.8	16.68±1.46	63	^***^	^***^	^***^
	14HS	20.4	26.8	23.81±0.98				
	15HS	18.8	24.3	21.96±1.04				
	14CP	19.6	25.1	22.62±1.07				
NDFD 48 h (NDF %)	13HS	11.0	20.2	16.11±1.46	76	^***^	^***^	^***^
	14HS	11.3	22.0	17.55±1.57				
	15HS	12.5	22.4	17.23±1.40				
	14CP	11.5	19.9	15.47±1.21				
ADL (DM %)	13HS	5.11	8.66	6.56±0.70	45.1	^***^	^***^	^***^
	14HS	2.70	5.64	4.46±0.47				
	15HS	3.27	6.10	5.00±0.38				
	14CP	2.88	5.44	4.26±0.44				

*E, environment; Min, minimum; Max, maximum; SD, standard deviation; G, genotype; H^2^, Broad-sense heritability*.

*Abbreviations of traits and E are explained in Materials and Methods*.

*** *Significant at P < 0.001*.

### Population structure and relative kinship

**Figure 1:**
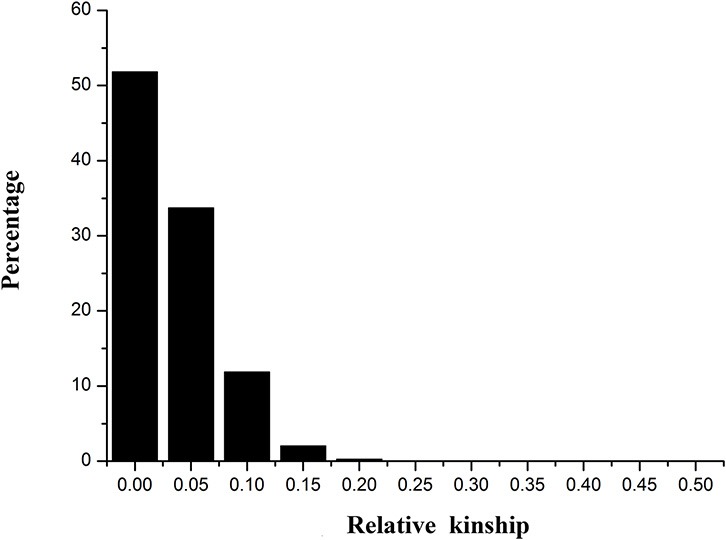
**Distribution of pairwise relative kinship estimates between 336 alfalfa genotypes**. The height of the black bar represents the percentage of genotypes in different ranges of kinships.

The genetic relationships among the genotypes were investigated using a model-based Bayesian clustering method on the 85 SSR marker genotyping data. Two populations were identified by STRUCTURE software using a Bayesian approach, corresponding to China, and the rest of the world as indicated by Qiang et al. (2015). The kinship was estimated based on the 85 SSR data on 336 alfalfa genotypes. About 51.8% of the pairwise kinship estimates were equal to 0, while 99.7% of the relative kinship estimates were < 0.2 in this alfalfa panel (Figure 1). These results indicated that most accessions have no or weak kinship with the other accessions in the panel, which might be due to the broad range collection of genotypes.

### Association analysis

**Figure 2:**
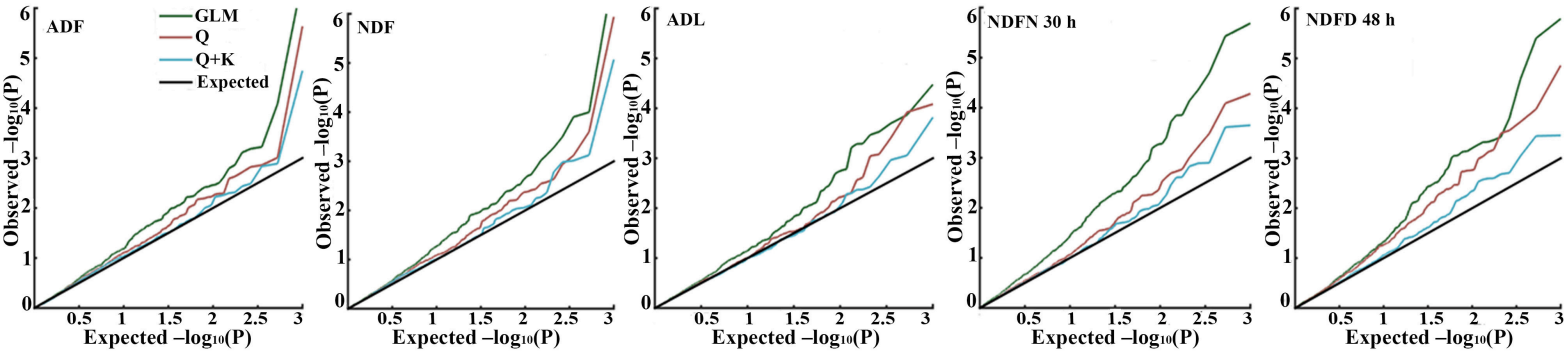
**QQ plot of observed vs. expected *P*-values using three different models for five fiber-related traits**. Cumulative distribution of *P*-values was computed from the SSR markers and phenotypes for the different association models. Data in environment 13HS of five traits was used in draw the QQ plot.

For all five fiber-related traits, association analyses were conducted to assess the performance of three different models (Table 2 and Figure 2). Generally, the observed *P*-value from GLM greatly deviated from the expected *P*-value, followed by the Q model, while the *P*-value from the Q + K model was close to the expected *P*-value (Table 2 and Figure 2). The result indicated that the false positives were well controlled in the MLM model in the study. Therefore, subsequent analyses were done based on the MLM model.

**Table 2:** **Association summary for five fiber-related traits using three models in different environments**.

**Traits**	**E**	**GLM**		**Q model**		**Q** + **K model**	
		**No of alleles**	***R*^2^ (%)**	**No of alleles**	***R*^2^ (%)**	**No of alleles**	***R*^2^ (%)**
ADF	13HS	16	2.5–8.4	7	2.8–6.8	6	2.5–6.0
	14HS	13	2.6–5.6	6	2.5–3.9	4	2.5–3.6
	15HS	16	2.6–5.2	12	2.5–3.6	12	2.5–3.6
	14CP	9	5.4–7.4	11	5.4–7.5	1	8.1
NDF	13HS	18	2.6–8.8	12	2.3–7.1	6	2.8–6.5
	14HS	14	2.7–5.5	10	5.5–7.7	1	8.28
	15HS	18	2.5–5.6	6	2.4–3.4	4	3.0–3.1
	14CP	9	5.5–7.4	10	2.4–3.7	9	2.9–3.8
NDFD 30 h	13HS	32	2.5–7.0	12	2.1–4.4	8	2.5–4.1
	14HS	9	2.6–4.2	8	5.5–6.9	2	5.9–7.0
	15HS	24	2.5–5.5	7	2.6–3.9	6	2.7–3.6
	14CP	12	5.6–7.4	12	2.5–5.7	8	2.9–6.0
NDFD 48 h	13HS	37	2.5–7.1	23	2.3–5.6	11	2.5–4.1
	14HS	18	2.5–5.7	10	6.1–9.2	10	5.8–9.3
	15HS	25	2.6–7.3	6	2.3–4.6	4	2.9–4.9
	14CP	11	5.4–9.4	16	2.4–4.5	13	2.6–4.0
ADL	13HS	9	2.5–5.2	8	2.5–5.1	5	2.6–5.3
	14HS	17	2.6–5.5	13	5.2–9.2	1	9.7
	15HS	30	2.6–5.1	8	2.5–4.6	7	2.6–4.5
	14CP	8	5.5–7.8	16	2.6–5.2	6	3.8–4.8

*Abbreviations of traits and E are explained in Materials and Methods*.

*R^2^, the explained phenotypic variance*.

Using the Q + K model, a total of 124 significant marker-trait associations was predicted under at least one environment (Table S2). For ADF trait associations, six, one, four, and 12 alleles were predicted as significant in 13HS, 14CP, 14HS, and 15HS data sets, respectively, with the explained phenotypic variance (*R*^2^) ranging from 2.48 to 8.13% (Table S2). For ADL, five, one, seven, and six associated alleles were identified in four environments, respectively, with the *R*^2^ varied from 2.56 to 9.66%. For NDF, six, one, four, and nine significant associated alleles were identified in four environments, respectively, with the *R*^2^ from 2.76 to 8.28%. For NDF 30 h, eight, two, six, and eight significant associated alleles were identified in four environments, respectively, with the *R*^2^ from 2.52 to 6.98%. For NDF 48 h, eight, eight, three, and 10 significant associated alleles were detected in four environments, respectively, with the *R*^2^ from 2.63 to 9.32%. Among these associated alleles, eight alleles were repeatedly observed in two environments (Table 3). For example, allele m13_173 associated with ADF was detected both in 14HS and 15HS. The allele m561-216 was associated with ADL both in 13HS and 14HS. In addition, among these associated alleles, 20 alleles were commonly associated with multiple fiber-related traits (Table S2). For example, the allele m561_216 was associated with ADF, ADL, NDF, NDF30, and NDF 48 h.

**Table 3:** **Summary of simple sequence repeat (SSR) alleles associated with fiber-related traits in at least two environments**.

**Trait**	**E**	**Associated alleles**	**Position**	**Chromosome**	***P*-value**	***R*^2^ (%)**	**Allele effects**
ADF	14HS	m13_173	221	2	0.00377	2.54	3.32
	15HS	m13_173	221	2	0.00058	3.60	3.32
	14HS	m350_342	113	1	0.00329	2.72	1.47
	15HS	m350_342	113	1	0.00276	2.73	1.29
	13HS	m561_216	406	3	0.00001	5.95	−1.48
	14HS	m561_216	406	3	0.00073	3.60	−1.35
ADL	13HS	m561_216	406	3	0.00005	5.25	−0.36
	14HS	m561_216	406	3	0.00428	2.56	−0.16
NDF	14HS	m13_173	221	2	0.00131	3.13	4.26
	15HS	m13_173	221	2	0.00047	3.76	3.84
	14HS	m350_342	113	1	0.00186	3.05	1.81
	15HS	m350_342	113	1	0.00146	3.12	1.56
	13HS	m561_216	406	3	0.00001	6.46	−1.92
	14HS	m561_216	406	3	0.00170	3.10	−1.45
NDFD 30 h	14HS	m350_342	113	1	0.00318	2.80	0.45
	15HS	m350_342	113	1	0.00002	5.96	0.69
NDFD 48 h	14CP	m115_183	913	7	0.00145	7.87	3.97
	15HS	m115_183	913	7	0.00278	2.71	3.71
	14HS	m13_173	221	2	0.00014	4.40	2.07
	15HS	m13_173	221	2	0.00153	2.97	1.43
	14CP	m5_152	517	4	0.00186	7.04	3.87
	15HS	m5_152	517	4	0.00235	2.77	3.67
	14CP	m520_134	791	7	0.00171	8.62	3.91
	15HS	m520_134	791	7	0.00241	2.99	3.67
	14CP	m520_137	793	7	0.00171	8.62	3.91
	15HS	m520_137	793	7	0.00241	2.99	3.67
	14HS	m561_216	406	3	0.00056	3.72	−0.65
	13HS	m561_216	406	3	0.00035	4.06	−0.63
	14HS	m710_236	663	5	0.00231	2.90	0.96
	15HS	m710_236	663	5	0.00303	2.63	0.84

*Abbreviations of traits and environments are explained in Materials and Methods*.

*R^2^, the explained phenotypic variance*.

The allele effect derived from significant marker-trait association was shown in Table S2. Among the markers associated with ADF, M115_183 had the most positive phenotypic effect (8.79), whereas m2_142 had the most negative phenotypic effect (−3.54). The alleles m215_182 and m2_142 had the most positive (9.95) and most negative (−4.11) phenotypic effect associated with NDF, respectively. For the NDFD 30 h, m190_205 had the most positive phenotypic effect (2.18), whereas m199_289 had the most negative phenotypic effect (−4.03). Among the alleles associated with NDFD 48 h, m225_203 had the most positive phenotypic effect (5.88), whereas m338_268 had the most negative phenotypic effect (−4.36). For the ADL, m53_131 had the most positive phenotypic effect (1.69), whereas m53_176 had the most negative phenotypic effect (−1.2). Also, m13, the individuals carrying the allele 170 bp had a lower ADL and NDFD 48 h than those carrying alleles 173 bp (Table S2). For m2, the individuals carrying the allele 136 bp had a lower NDFD48h than those carrying alleles 140 bp (Table S2). For m225, the individuals carrying the allele 191 bp had a lower NDFD48h than those carrying alleles 203 bp (Table S2). For m53, the individuals carrying the allele 176 bp had a lower ADL than those carrying alleles 131 bp (Table S2).

## Discussion

Association mapping has increasingly become a viable approach for the genetic dissection of quantitative traits. Due to the diverse geographical origins, the germplasm panel may contain either population structure or familial relatedness (Yu and Buckler, 2006). One of the limitations of association mapping studies is the easy detection of false positives associations caused by the existence of the genetic structure in the populations studied (Flint-Garcia et al., 2005). Several researches reported, in the structured association population, the mixed model (Q + K) showed a significant improvement in goodness of fit for traits (Flint-Garcia et al., 2005; Yu et al., 2006). In this panel, association analysis was conducted for five fiber-related traits in four environments using the GLM−simple model, the Q model, and the Q + K model. The alfalfa association populations used in this study contained population structure but no obvious familial relationships (Figure 1). The quantile–quantile (QQ) plots indicated that the Q + K model performed best for all five fiber-traits, It seems that the Q + K model was sufficient to minimize false-positive associations, especially for some traits not influenced by population structure, which was consistent with other model simulations and comparisons (Yu et al., 2006; Zhu et al., 2008). All the results indicate that model testing for quantitative traits is necessary for increasing the accuracy of association.

There was no previous study on alfalfa fiber trait mapping or association using molecular markers. A total of 124 alleles from 38 markers accounted for phenotypic variation with 2.46–9.66% were identified as associated with five fiber-related traits based on association analysis using the Q + K model (Table S2). These associated alleles were not consistent with the previous studies of Li et al. (2011) which may be explained by the different markers and different population used in the two studies. These was also observed in previous studies on linkage mapping and association mapping which found that different mapping populations detected different QTL regions (Agrama et al., 2007; Zhang et al., 2014).

Most of the loci that were associated with the five traits could only be identified in a specific environment, indicating that the fiber-related traits in the study are variously influenced by the environment. However, some stable associations were identified in our study, such as the allele m350_342 which located in chromosome 1 were repeatedly detected in two environments and associated with ADF, NDF, NDFN30h, and NDFD48h. Three markers, m115_183, m520_134, and m520_137, located in chromosome 7 were repeatedly detected in two environments and associated with NDFD48h. Markers with significant traits associated over multiple environments may indicate that the associated genes are more stably expressed (i.e., less environmental influence) (Ray et al., 2015). A low threshold, *P* < 0.005, was used to detect the marker-trait association due to the limited number of marker used in this study. If high-density DNA polymorphism datasets are used for association mapping, additional markers with high –Log (*P*-value) may be obtained.

Among these associated alleles, different distributed patterns were observed among eight chromosomes in alfalfa. Eight alleles from seven markers which associated all five traits were observed in the chromosome 1, while only one allele of one marker which associated two traits was observed in the chromosome 6 (Table S2). In the study, 20 markers were associated with more than one traits indicated these traits were correlative each other. Interestingly, the markers, m225, and m338, reportedly associated with yield (Li et al., 2011), was found associated with NDFD 30h and NDFD48h in this study, suggesting a correlation between these traits as assessed by the SSR or these traits are controlled by the same or neighboring regions in the genome. The explained phenotypic variance of all associated alleles ranged from 2.46 to 9.66%, with mean of 3.84%. The result indicated that the fiber-related traits were complex in nature, i.e., controlled by multiple genes without obvious major effects.

The present study is the first attempt in associating alfalfa fiber-related traits with the genotyping results derived from SSR markers using a diverse set of global collection of alfalfa genotypes. Our results demonstrated that this alfalfa panel is suitable for association mapping analysis targeting complex quantitative traits with optimal association models. The markers associated to the QTLs in the study can be effectively used in further alfalfa marker assisted breeding programmers for introgression of alleles into locally well adapted germplasm.

## Author contributions

ZW designed the experiments performed the statistical analysis and drafted the manuscript. HQ performed SSR genotyping. HZ, GL, ZZ, RX, and YZ conducted the quality analysis. XW and HG revised manuscript. All authors have read and approved the final manuscript.

### Conflict of interest statement

The reviewer HC declared a shared affiliation, though no other collaboration, with the authors RX and YZ to the handling Editor, who ensured that the process nevertheless met the standards of a fair and objective review. The other authors declare that the research was conducted in the absence of any commercial or financial relationships that could be construed as a potential conflict of interest.

## Supplementary material

Table S1 **Accession No. origin, improvement status, cultivar name, and No. of genotypes sampled of 336 alfalfa genotypes**.

Table S2 **List of associated SSR alleles of five fiber-related traits in different environments**.

Figure S1 **Histograms showing frequency distribution of five fiber-related traits in different environments in the study**. The y-axis denotes the value of frequency, whereas the x-axis shows resultant groups of genotypes.
