# Supplementary figures and images for: Association Mapping for Fiber-Related Traits and Digestibility in Alfalfa (Medicago sativa)
Source: Front Plant Sci. 2016 Mar 18;7:331. doi: 10.3389/fpls.2016.00331 (PMC4797558; doi:10.3389/fpls.2016.00331)

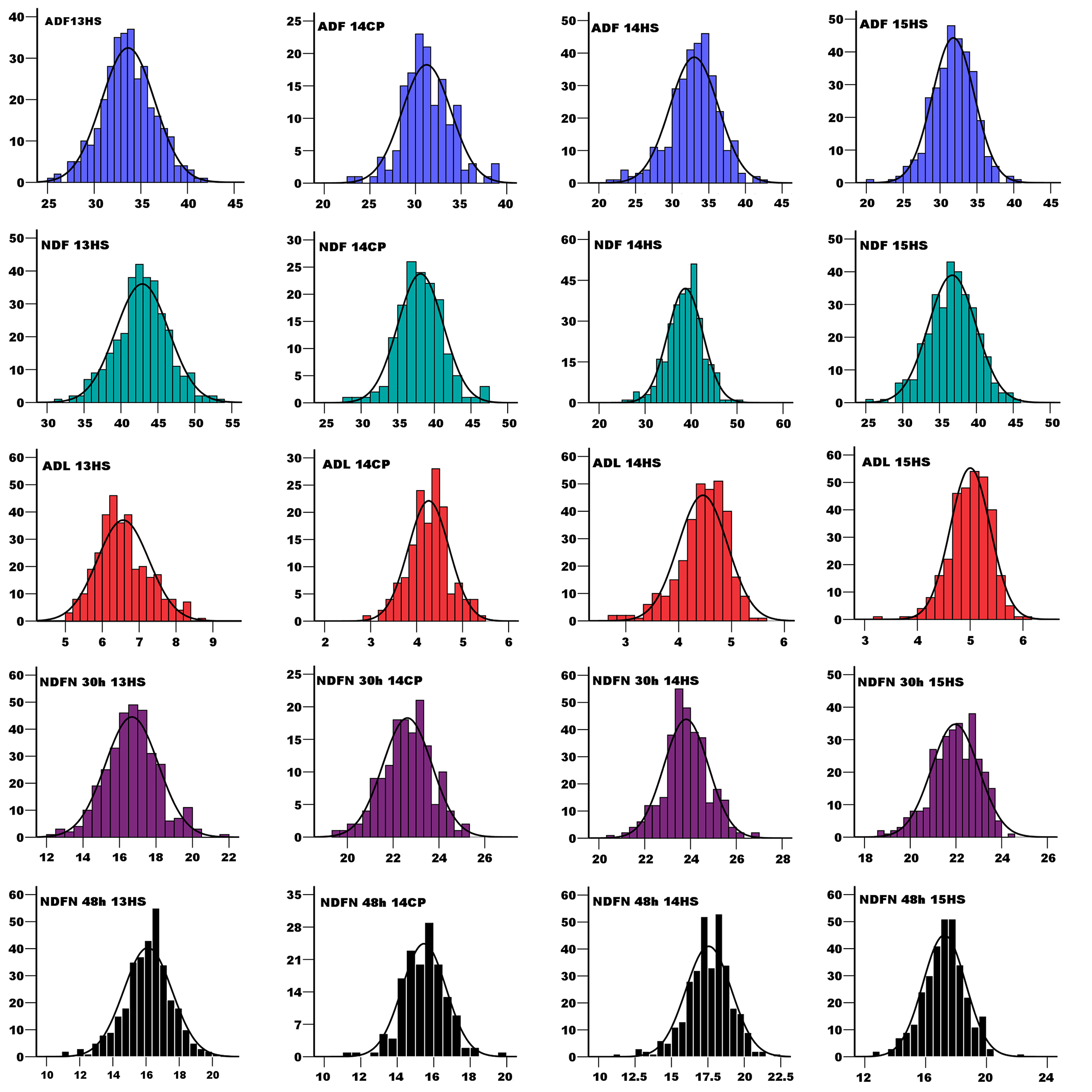

Supplement: Figure S1 — Histograms showing frequency distribution of five fiber-related traits in different environments in the study. The y-axis denotes the value of frequency, whereas the x-axis shows resultant groups of genotypes. [file Image1.TIF]
